# A Screen for Kinetochore-Microtubule Interaction Inhibitors Identifies Novel Antitubulin Compounds

**DOI:** 10.1371/journal.pone.0011603

**Published:** 2010-07-15

**Authors:** Emanuela Screpanti, Stefano Santaguida, Tam Nguyen, Romano Silvestri, Rick Gussio, Andrea Musacchio, Ernest Hamel, Peter De Wulf

**Affiliations:** 1 Department of Experimental Oncology, European Institute of Oncology, IFOM-IEO Campus, Milan, Italy; 2 Information Technology Branch, Developmental Therapeutics Program, Division of Cancer Treatment and Diagnosis, National Cancer Institute at Frederick, National Institutes of Health, Frederick, Maryland, United States of America; 3 Department of Medicinal Chemistry and Technologies, University of Rome “La Sapienza”, Rome, Italy; 4 Toxicology and Pharmacology Branch, Developmental Therapeutics Program, Division of Cancer Treatment and Diagnosis, National Cancer Institute at Frederick, National Institutes of Health, Frederick, Maryland, United States of America; Virginia Tech, United States of America

## Abstract

**Background:**

Protein assemblies named kinetochores bind sister chromatids to the mitotic spindle and orchestrate sister chromatid segregation. Interference with kinetochore activity triggers a spindle checkpoint mediated arrest in mitosis, which frequently ends in cell death. We set out to identify small compounds that inhibit kinetochore-microtubule binding for use in kinetochore-spindle interaction studies and to develop them into novel anticancer drugs.

**Methodology/Principal Findings:**

A fluorescence microscopy-based *in vitro* assay was developed to screen compound libraries for molecules that prevented the binding of a recombinant human Ndc80 kinetochore complex to taxol-stabilized microtubules. An active compound was identified that acted at the microtubule level. More specifically, by localizing to the colchicine-binding site in αβ-tubulin the hit compound prevented the Ndc80 complex from binding to the microtubule surface. Next, structure-activity analyses distinguished active regions in the compound and led to the identification of highly potent analogs that killed cancer cells with an efficacy equaling that of established spindle drugs.

**Conclusions/Significance:**

The compound identified in our screen and its subsequently identified analogs represent new antitubulin chemotypes that can be synthetically developed into a novel class of antimitotic spindle drugs. In addition, they are stereochemically unique as their *R-* and *S-*isomers mimic binding of colchicine and podophyllotoxin, respectively, two antitubulin drugs that interact differently with the tubulin interface. Model-driven manipulation of our compounds promises to advance insight into how antitubulin drugs act upon tubulin. These advances in turn may lead to tailor-made colchicine site agents which would be valuable new assets to fight a variety of tumors, including those that have become resistant to the (antispindle) drugs used today.

## Introduction

Many anticancer drugs used in the clinic inhibit cell division as tumors are characterized by uncontrolled proliferation [1]. Cell division is the process during which a mother cell generates two genetically identical daughter cells. In S phase, maternal chromosomes replicate and form sister chromatid pairs. During the subsequent M phase, protein assemblies called kinetochores form on the centromere of each chromatid and attach the sister chromatids in a bipolar manner to the microtubules (MTs) of the mitotic spindle. The spindle MTs are a dynamic array of αβ-tubulin fibers that extend from two oppositely localized centrosomes. At the metaphase-anaphase transition, the sister chromatids are first separated and then segregated into the daughter cells. During the final cell cycle stage named cytokinesis, the daughters divide, each containing an identical set of chromosomes [2].

Antiproliferative drugs used in the clinic include agents that target mitotic spindle integrity or dynamics [3]. In response to the spindle defects caused by these drugs, the spindle assembly checkpoint (SAC) delays mitosis allowing cells to reverse the drug-induced damage [4]. Cells that do not recover and satisfy the SAC either undergo cell death (apoptosis) or adapt. Adapting cells may continue to cycle, undergo senescence or die in the subsequent interphase [5]. Almost all antispindle drugs suppress MT integrity and dynamics by stabilizing MTs and stimulating tubulin polymerization, or by destabilizing MTs and inhibiting tubulin polymerization. MT stabilizing drugs including taxanes and ixabepilone, or MT destabilizing agents including vinca alkaloids and estramustine, are very effective against a broad range of tumors [3]. However, resistance to antitubulin drugs has become a significant problem due to P-glycoprotein overexpression and, perhaps, to mutations in genes encoding the tubulin subunits, changes in tubulin isotype composition of MTs, altered expression or binding of MT-regulatory proteins including Tau, mutations in or reduced levels of γ-actin, and/or a reduced apoptotic response [3], [6]. To deal with resistance, structurally diverse antiMT drugs are being developed while alternative mitosis-specific drug targets are being evaluated [7], [8].

A mitosis-specific structure that has recently been focused on for development into a drug target is the kinetochore, the protein complex that coordinates chromosome segregation. Interfering with kinetochore activities, including MT binding, triggers a SAC-mediated arrest of mitosis, which frequently leads to cell death [9]. As kinetochores assemble from >100 proteins, they are, in principle, almost inexhaustible drug targets.

We wished to identify compounds that inhibit kinetochore-MT binding to develop them into new antimitotic agents. We also wanted to use these compounds as chemobiological tools to study the mechanisms that drive kinetochore-MT binding. To identify such compounds we focused on the outer kinetochore Ndc80 complex, which attaches the kinetochore structure to the MTs of the mitotic spindle [10]. To screen chemical libraries for active molecules we developed an *in vitro* fluorescence microscopy-based binding assay using a recombinant Ndc80 complex and taxol-stabilized MTs. Of 10,200 compounds screened, one compound prevented the Ndc80 complex from binding to the MTs by acting at the MT level. More specifically, the compound localized to the colchicine-binding site at the αβ-tubulin interface. Using a computational approach, the antitubulin compound was structurally dissected and analogs were identified containing a 20-fold higher antitubulin activity. Of these, the most potent compound mitotically arrested and killed adenocarcinoma cells with an IC_50_ value of 25 nmol/l.

The classic colchicine site agents (e.g. colchicines, combretastatins, podophyllotoxin), most of which are structurally similar and rather complex in nature, are not used in the clinic because they are systemically toxic. This is unfortunate as colchicine site agents would represent powerful alternatives to the clinically used taxane- or vinca-site drugs against which tumor cells have been developing resistance. Structurally novel or less complicated colchicine site compounds may be the answer to the problem of toxicity, as illustrated by the highly potent stilbene colchicine derivatives, which exhibit minimal toxicity [11]. The antitubulin hit compound and lead analogs identified in this study are chemotypically unique colchicine site agents. In addition, they interact with the colchicine-binding pocket in a unique manner: our docking studies suggest that the *R*-isomers interact with tubulin via their furan ring, while the *S*-isomers localize to the colchicine pocket via their ester side chain. Future analysis and modification of our compounds will advance insight into the colchicine site-drug interaction and promise to result in new anticancer compounds with optimal performance and, possibly, minimal toxicity.

## Results

### Microscopy-based *in vitro* screen for compounds that prevent binding of the Ndc80 kinetochore complex to MTs

**Figure 1:**
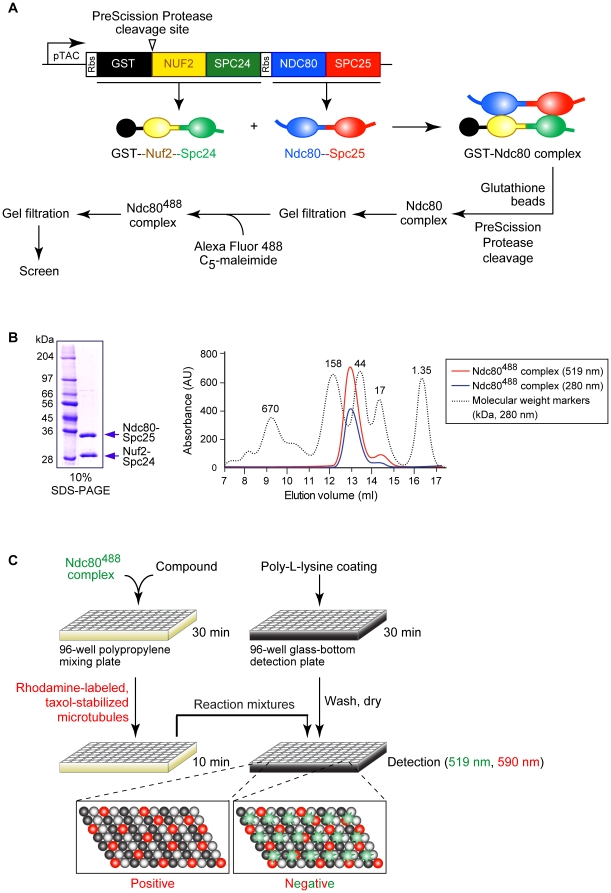
Screen for compounds inhibiting binding of the recombinant Ndc80 kinetochore complex to taxol-stabilized microtubules. Schematic of the expression, purification and fluorescent labeling of the recombinant human Ndc80 kinetochore complex (Ndc80^488^ complex) used in the screen (***A***). Recombinant Ndc80 complex obtained by GST-based purification and PreScission Protease-mediated release from the affinity beads (left panel). Gel filtration profile (Superdex 200) of the Ndc80^488^ complex obtained by labeling the purified complex with Alexa Fluor 488 C_5_-maleimide fluorophore (right panel) (***B***). Schematic of the fluorescence microscopy-based screen for compounds inhibiting the binding of the Ndc80^488^ complex (green) to rhodamine (red) labeled, taxol-stabilized MTs (tubulin subunits are colored black and grey) (***C***).

**Figure 2:**
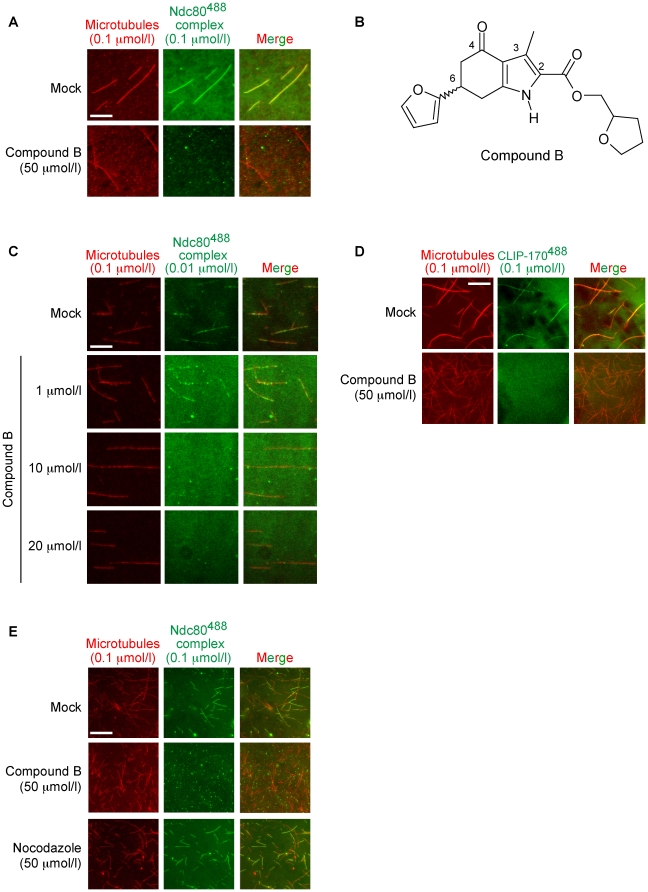
Compound B prevents the recombinant Ndc80 kinetochore complex from binding to taxol-stabilized microtubules. Effect of compound B (50 µmol/l) on binding of the Ndc80^488^ complex to rhodamine-labeled, taxol-stabilized MTs as assayed using inverted wide-field fluorescence microscopy. The scale bar represents 4 µm (***A***). Structure of compound B; 6-furan-2-yl-3-methyl-4-oxo-4,5,6,7-tetrahydro-1*H*-indole-2-carboxylic acid (tetrahydro-furan-2-yl)methyl ester (***B***). Measurement of the IC_50_ value of compound B using total internal reflection fluorescence microscopy. The scale bar represents 4 µm (***C***). Compound B (50 µmol/l) prevented MT-associating protein CLIP-170^488^, used as a specificity control, from binding to taxol-stabilized MTs. The scale bar represents 4 µm (***D***). Antitubulin control drug nocodazole (50 µmol/l) did not prevent the Ndc80^488^ complex from binding to MTs. The scale bar represents 4 µm (***E***).

To screen compound libraries for molecules inhibiting binding of the outer kinetochore Ndc80 complex (Ndc80, Nuf2, Spc24, Spc25) to MTs, we used a fluorescence microscopy-based *in vitro* approach (Fig. 1). First, the recombinant human Ndc80 construct, used to crystallize the complex [12], was produced in *Escherichia coli* using a bicistronic plasmid from which the Nuf2-Spc24 and Ndc80-Spc25 peptides were generated (Fig. 1A). Following their intracellular assembly, the complex was purified from *E. coli* cell extract based on the GST tag at the N-terminus of Nuf2. The complex was released in solution with PreScission Protease (its recognition sequence was introduced between the GST tag and the *NUF2* coding sequence) and was separated from contaminants by gel filtration chromatography (Fig. 1A). The high degree of purity of the preparation was confirmed by SDS-PAGE analysis and coomassie staining, which identified only both peptides (Fig. 1B). Next, the Ndc80 complex was fluorescently labeled with the Alexa Fluor 488 C_5_-maleimide (next named the Ndc80^488^ complex) and was separated from unreacted fluorophore by gel filtration chromatography. The final preparation was highly pure as evidenced by gel filtration analysis and detection at 519 nm (emission maximum of the Alexa Fluor 488 dye) and at 280 nm (intrinsic protein fluorescence) (Fig. 1B). Next, the Ndc80^488^ complex (0.1 µmol/l) was incubated with 10,200 compounds (comprising the Chembridge DIVERset and a compound collection from the Univ. Rome) at a starting concentration of 50 µmol/l. Binding (or lack thereof) of the complex to rhodamine-labeled taxol-stabilized MTs (0.1 µmol/l) was scored by wide-field fluorescence microscopy (Fig. 1C; images in Fig. 2A). One compound, named “compound B” (6-furan-2-yl-3-methyl-4-oxo-4,5,6,7,-tetrahydro-1*H*-indole-2-carboxylic acid tetrahydro-furan-2-ylmethyl ester; DIVERset, Chembridge) was found to be active (Fig. 2A,B). Total internal reflection fluorescence microscopy was used to establish the IC_50_ value of the compound, which was measured to be ∼20 µmol/l (Fig. 2C).

### Functional evaluation of the inhibition of Ndc80^488^ complex-MT binding by compound B

A compound inhibiting the Ndc80^488^ complex-MT interaction could act either against the complex or against the MTs. To probe the specificity of compound B toward the Ndc80^488^ complex, we examined its ability to inhibit MT binding of recombinant CLIP-170^488^, a MT plus-end tracking protein. We found that compound B prevented CLIP-170^488^ from binding to MTs (Fig. 2D), suggesting that compound B acted at the MT level. Of note, MT poison nocodazole did not prevent the Ndc80^488^ complex from binding to the taxol-stabilized MTs (Fig. 2E), suggesting that compound B interacted in a unique manner with the stabilized MT polymer, and indirectly, with binding of the Ndc80^488^ complex to the MTs.

**Figure 3:**
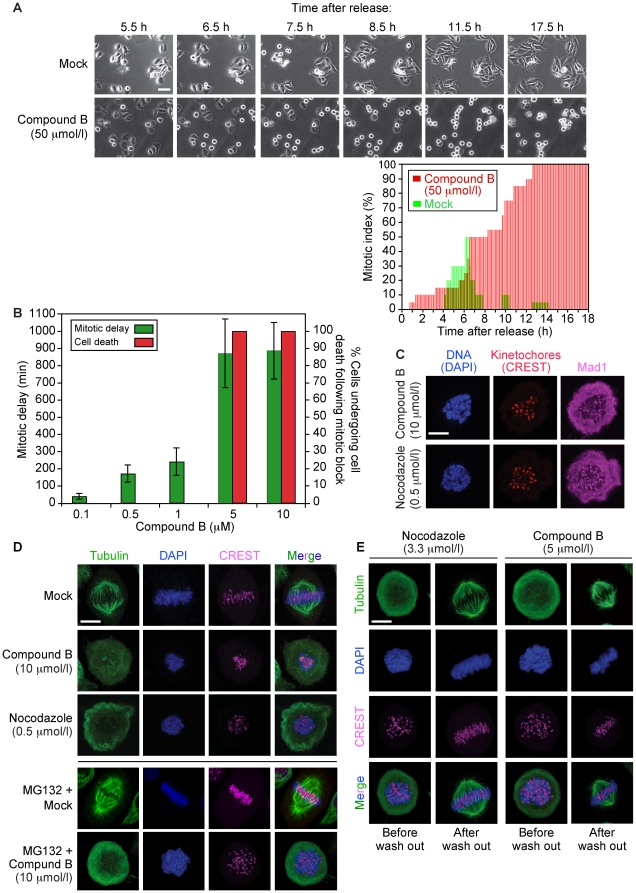
Compound B inhibits tubulin assembly and destabilizes spindles, resulting in mitotic block and cell death. Live-cell imaging of HeLa cells synchronized in G1/S and released into medium containing compound B (50 µmol/l) or mock solution (1% DMSO). The scale bar represents 20 µm. The plot shows the mitotic index of the cultures over time (compound B, red bars; mock [1% DMSO], green bars) (***A***). Compound B induced a mitotic delay (1–10 µmol/l) and cell death (5–10 µmol/l) (***B***). The mitotic delay triggered by compound B (10 µmol/l) resulted from mitotic checkpoint activity as indicated by the presence of SAC protein Mad1 at kinetochores. Nocodazole (0.5 µmol/l) acted as a positive control. The scale bar represents 10 µm (***C***). Compound B inhibited spindle formation (row 2) and destabilized metaphase spindles (row 5) in HeLa cells. Nocodazole (0.5 µmol/l) acted as the positive control. The scale bar represents 10 µm (***D***). The antitubulin activity of compound B on human cells is reversible as the cells formed a mitotic spindle structure when the drug was washed out and the cells were released into fresh medium containing 10 µmol/l MG132. Nocodazole (0.5 µmol/l) acted as the positive control. The scale bar represents 10 µm (***E***).

To study whether compound B affected mitosis, HeLa cells were synchronously released from G1/S (thymidine block) into growth media containing 0.1–50 µmol/l compound B. Time-lapse videomicroscopy showed an accumulation of mitotic cells in the presence of the compound, while the mock-treated cells progressed through mitosis (Fig. 3A, *upper and lower panels*). At compound B concentrations of 5 µmol/l and above, the cells arrested robustly in metaphase (∼15 h) and then underwent cell death, as diagnosed by cell shrinkage (Fig. 3B). The observed mitotic delay came from mitotic checkpoint activity as confocal immunofluorescence (IF) imaging showed that SAC protein Mad1 accumulated at kinetochores in cells treated with compound B (as with nocodazole, which was used as a positive control, Fig. 3C). The IF analysis further revealed that sister chromatids (DAPI) and kinetochores (CREST serum) were not aligned on the metaphase plate. This phenotype is indicative of chromatids being unable to bind to spindle MTs and/or of spindle defects, as observed with nocodazole (Fig. 3C). To determine whether compound B affected kinetochore-spindle attachment or interfered with spindle integrity, we examined by confocal IF imaging the localization of chromosomes and kinetochores, and the state of the spindle (anti-α-tubulin) in cells synchronously released from a G1/S arrest into medium containing 10 µmol/l of compound B. All cells lacked a mitotic spindle, as with nocodazole (Fig. 3D), supporting the idea that compound B acts at the MT level, probably by inhibiting tubulin assembly. Because drugs that inhibit tubulin polymerization also destabilize MTs [3], we next probed whether compound B destabilized metaphase spindles. We arrested HeLa cells in metaphase using 10 µmol/l of proteosome inhibitor MG132. The cells, all of which contained a mitotic spindle, were then treated with 1% DMSO or 10 µmol/l compound B. IF imaging showed that compound B depolymerized the spindle (Fig. 3D, *bottom set of panels*). Thus, compound B prevents tubulin assembly and destabilizes spindle MTs in cells.

To probe whether the activity of compound B is reversible or not, we synchronously released G1/S arrested HeLa cells into fresh medium containing 5 µmol/l compound B (nocodazole was used as the positive control). The cells efficiently arrested in metaphase due to absence of a mitotic spindle (Fig. 3E, *left column of images in each set*). Compound B and nocodazole were then washed out and the cells were released in MG132 containing medium. Within 3 h, all cells had arrested with a mitotic spindle suggesting that our compound does not covalently bind to tubulin, allowing for full reversibility of its intracellular activity (Fig. 3E, *right column of images in each set*).

### Taxol inhibits the MT-depolymerization activity of compound B

**Figure 4:**
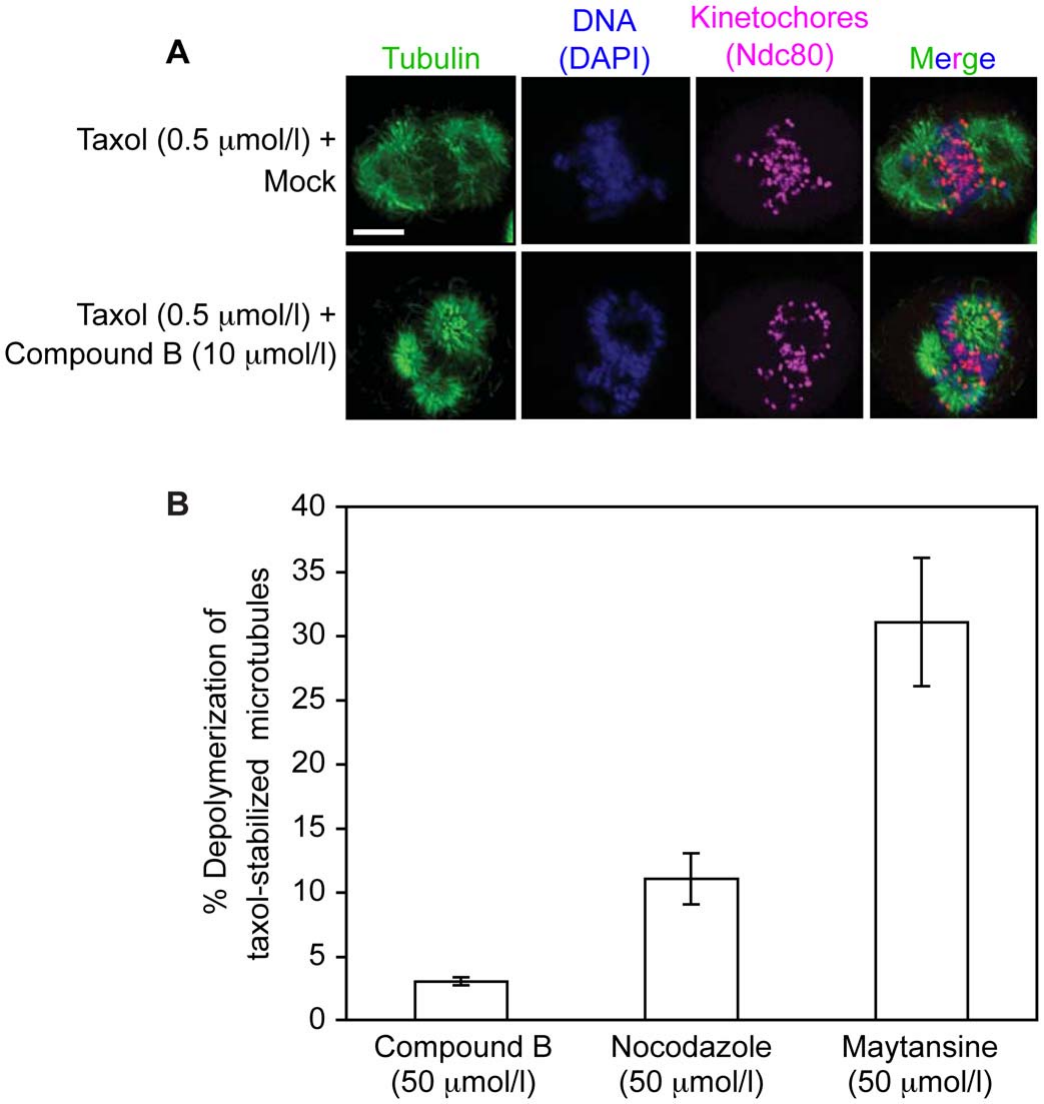
Taxol inhibits the microtubule-destabilizing activity of compound B. Taxol (0.5 µmol/l) prevented spindle depolymerization by compound B (10 µmol/l) when both were added to HeLa cells arrested in metaphase with proteosome inhibitor MG132. The scale bar represents 10 µm (***A***). Depolymerization by compound B, nocodazole, and maytansine (50 µmol/l each) of taxol-stabilized MTs *in vitro*. The bars represent standard errors (***B***).

During our screen we found MTs to be stable in the presence of compound B (Fig. 2, *panel A and panels C to E*), suggesting that taxol prevented MT depolymerization by the compound. To test this hypothesis, we treated HeLa cells with 0.5 µmol/l taxol, thereby arresting them in metaphase with stabilized spindles. Following the addition of either 10 µmol/l compound B or 1% DMSO, the spindles did not depolymerize (Fig. 4A). Hence, the effects of taxol are likely dominant over those of compound B since our previous experiments had shown that compound B alone efficiently depolymerized metaphase spindles at 10 µmol/l (Fig. 3, *panels C and D*). To quantify the taxol-mediated inhibition of MT disassembly by compound B, we measured *in vitro* the depolymerization of taxol (20 µmol/l)-stabilized MTs following incubation with 50 µmol/l compound B (the screen condition). Nocodazole and maytansine (both at 50 µmol/l) were included as controls. At 50 µmol/l, compound B depolymerized 3% of the taxol-stabilized MT population, while nocodazole and maytansine depolymerized 11% and 31% of the taxol-treated MTs, respectively (Fig. 4B). The limited ability of compound B to depolymerize taxol-stabilized MTs explains why we did not identify compound B as a MT-depolymerizing drug in our screen. This finding further supports our hypothesis that the interaction of compound B with the stabilized MTs must have prevented the Ndc80^488^ complex from establishing contact with the MT surface.

### Compound B is a colchicine site effector of MT assembly and stability

**Figure 5:**
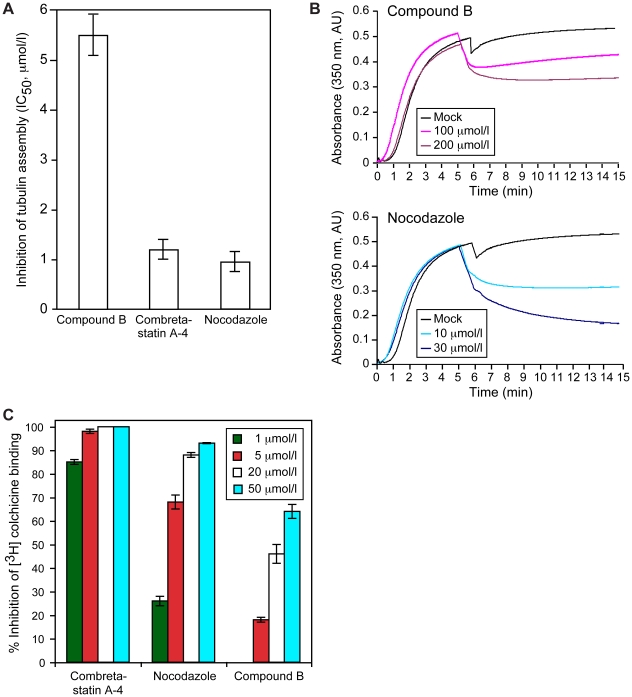
Compound B inhibits tubulin assembly and destabilizes MTs by acting at the tubulin colchicine site. Compound B inhibited tubulin assembly *in vitro*; combretastatin A-4 and nocodazole acted as positive controls (***A***). Compound B depolymerized non-stabilized MTs *in vitro*; nocodazole acted as the positive control (***B***). Compound B competed with [^3^H]colchicine for binding to the colchicine site in αβ-tubulin. The bars represent standard deviations (***C***).

To quantify the antitubulin activity of compound B, we added 1–50 µmol/l compound B to 10 µmol/l αβ-tubulin. Tubulin polymerization in GTP-containing buffer was tracked turbidimetrically at 350 nm [13], [14], with combretastatin A-4 and nocodazole acting as controls. Compound B inhibited tubulin polymerization with an IC_50_ value of 5.5 µmol/l, whereas combretastatin A-4 and nocodazole were 5-fold more effective (IC_50_ values of 1.2 µmol/l and 0.96 µmol/l, respectively; Fig. 5A). Because compound B also destabilized spindle MTs in cells (Fig. 3D), we also quantified *in vitro* the MT depolymerization activity of the compound. For this purpose, MTs were assembled from 10 µmol/l αβ-tubulin; 5 min into the reaction compound B was added in concentrations up to 200 µmol/l. MT depolymerization was tracked as a decrease in turbidity at 350 nm (Fig. 5B). Compound B affected MT stability only at molar ratios exceeding 20∶1, while nocodazole induced MT depolymerization at a drug:tubulin ratio of 1 (Fig. 5B). Thus, compound B inhibited tubulin polymerization at a concentration at least 40-fold lower than that needed to disassemble MTs. In contrast, a 10-fold difference was observed for nocodazole (Fig. 5B versus Fig. 5A). The measured discrepancies in the antitubulin activities of compound B are typical for tubulin destabilizing drugs [3].

Agents that inhibit tubulin polymerization and destabilize MTs include the colchicinoids, combretastatins, benzimidazole carbamates, vinca alkaloids, and maytansinoids [3]. These drugs act primarily at β-tubulin in the αβ-heterodimer, albeit at different sites. Colchicinoids, combretastatins and benzimidazole carbamates (e.g. nocodazole) localize to the colchicine site near the C-terminus of β-tubulin at the αβ-tubulin intradimer interface [15], [16], whereas vinca alkaloids and maytansinoids localize to the N-terminal GTPase domain of β-tubulin [17], [18]. To determine whether compound B (1–50 µmol/l) acts at the colchicine-binding site, we assayed whether it inhibited binding of [^3^H]colchicine (5 µmol/l) to tubulin (1 µmol/l), in comparison with combretastatin A-4 and nocodazole (Fig. 5C). Compound B significantly inhibited [^3^H]colchicine binding to tubulin, indicating that it acts at the colchicine site. Its binding affinity, however, was ∼3-fold lower than that of nocodazole (compare values at 5 µmol/l and 20 µmol/l, Fig. 5C).

### Structure-activity analysis of compound B leads to the identification of furan-metoticas

**Figure 6:**
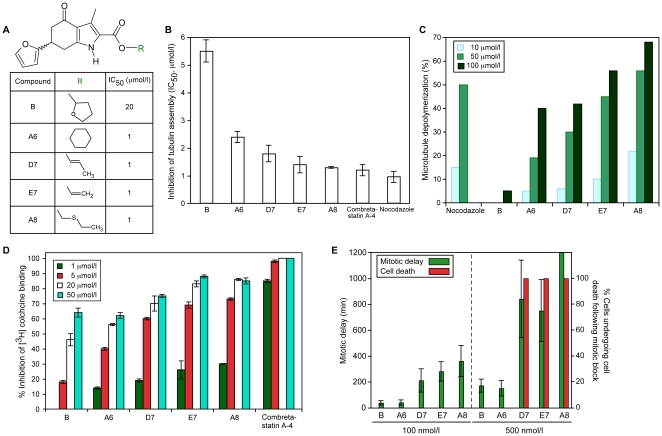
Furan-metoticas inhibit tubulin assembly and destabilize microtubules. Structures of furan-metoticas B, A6, D7, E7, and A8, and their IC_50_ values measured in the Ndc80^488^-MT binding assay (***A***). Compounds A6, D7, E7 and A8 inhibited tubulin polymerization *in vitro* with an efficiency exceeding that of compound B; combretastatin A-4 and nocodazole acted as positive controls. The bars represent standard deviations (***B***). Compounds A6, D7, E7 and A8 efficiently depolymerized non-stabilized MTs *in vitro*; nocodazole acted as a positive control (***C***). Compounds B, A6, D7, E7 and A8 competed with [^3^H]colchicine for binding to tubulin; combretastatin A-4 acted as the positive control. The bars represent standard deviations (***D***). Furan-metoticas triggered a mitotic block and subsequent cell death with high efficiency in HeLa cells (cervical adenocarcinoma). The bars represent standard deviations (***E***).

Compound B (Fig. 2B) represents a novel antitubulin chemotype. Consequently, its overt lack of structural similarity with known colchicine site agents made it difficult to understand which part of compound B was responsible for its antitubulin activity. To solve this problem, we performed a computer-assisted search of commercial libraries comprising ∼3,000,000 compounds for molecules containing either a 6-furan-2-yl-3-methyl-4-oxo-4,5,6,7-tetrahydro-1*H*-indole structure (encircled in blue, Fig. S1) or the C2 carboxylate-based tetrahydrofurfuryl side group (encircled in red, Fig. S1). We identified 104 compounds containing a tetrahydrofurfuryl group; none were active in the Ndc80^488^ complex-MT binding assay. In contrast, 17 compounds containing the 6-furan-2-yl-3-methyl-4-oxo-4,5,6,7-tetrahydro-1*H*-indole fragment inhibited to varying extent the binding of the Ndc80^488^ complex to MTs, suggesting that this region harbors the antitubulin activity of compound B. Four of the 17 compounds (named A6, D7, E7, and A8) were ∼20-fold more active than compound B, as measured in the Ndc80^488^ complex-MT binding assay (IC_50_ values of ∼1 µmol/l; Fig. 6A). Their enhanced activity was also confirmed turbidimetrically: the IC_50_ values of compounds A6, D7, E7 and A8 for inhibiting tubulin assembly were 2.3, 3.0, 4.0, and 4.2-fold lower, respectively, than that of compound B. The activity of the most potent molecule, A8, was similar to that of combretastatin A-4 and nocodazole (Fig. 6B). The capacity of these four molecules to destabilize MTs *in vitro* was also superior to that of compound B; the ability of compound A8 to depolymerize MTs was 12-fold higher than that of compound B and was 10% higher than that of nocodazole (Fig. 6C). The high antitubulin activity of compounds D7, A6, A8 and E7 likely results from an improved ability of the compounds to localize to the colchicine site. Indeed, compounds D7, A6, A8 and E7 were 3-fold more active than compound B as inhibitors of [^3^H]colchicine-tubulin binding (Fig. 6D), concordant with their inhibitory effects on tubulin assembly (Fig. 6B).

Next, we determined whether the elevated antitubulin activities of compounds A6, A8, D7 and E7 (versus compound B), and the discrepancies in activity between the compounds themselves were reflected in their effects on mitosis. G1/S synchronized HeLa cells were released into growth medium containing 100 or 500 nmol/l of these compounds or compound B; the duration of the mitotic delay by the compounds and the percentage of cells undergoing subsequent cell death were scored. Except for A6, the new compounds were also more active than compound B in this cell-based assay (Fig. 6E). Possibly, compound A6, which has a bulky side group similar to that of compound B (Fig. 6A) has a reduced uptake or lower affinity for the colchicine site in cells. The four compounds triggered mitotic delays to an extent that directly reflects their activities *in vitro* (compare Fig. 6E and Fig. 6, *panels B to D*). The observation that the compounds caused mitotic delays of varying duration also suggests that their side chains are important for their biological activity and are unlikely to be hydrolyzed in human cells. At 500 nmol/l, the most potent molecules (D7, E7, and A8) elicited a robust mitotic block, followed by cell death (Fig. 6E). Of note, each of our compounds acted in cells with an efficacy exceeding those measured *in vitro* (compare Fig. 6E and Fig. 5). This discrepancy may be due to increased compound:tubulin ratios in cells. Alternatively, the compounds may be modified intracellularly, resulting in enhanced activity. Because compound B and the four second-generation compounds are 6-furan-2-yl-3-methyl-4-oxo-4,5,6,7-tetrahydro-1*H*-indole-2-carboxylic acid derivatives, we have named these tubulin inhibitors “furan-metoticas”.

### Antiproliferation activity of furan-metoticas

**Figure 7:**
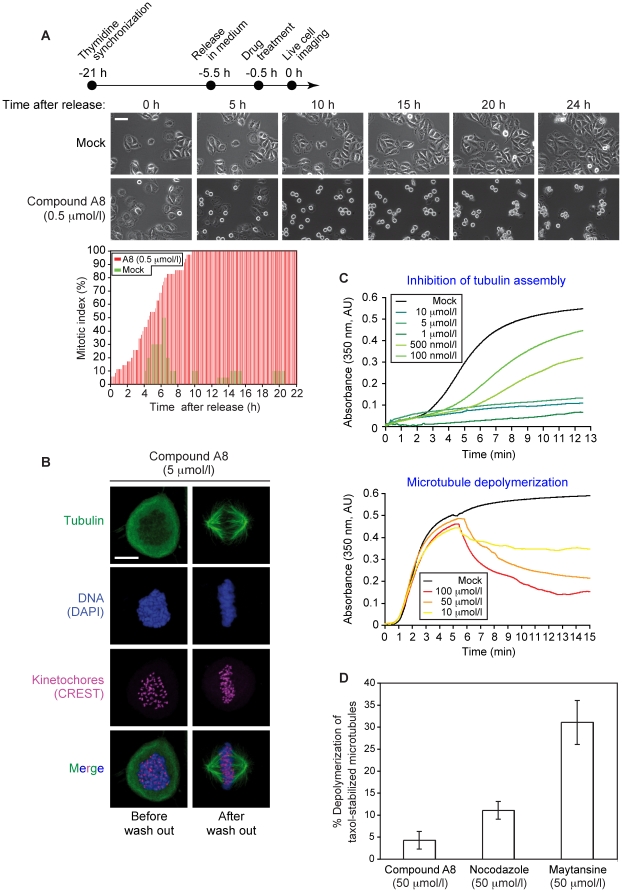
Compound A8 inhibits tubulin assembly and destabilizes spindle microtubules in HeLa cells and *in vitro*. Live-cell videomicroscopy of HeLa cells treated with mock solution (1% DMSO) or with compound A8 (0.5 µmol/l) following release from synchronization in G1/S (upper panel). The scale bar represents 20 µm. The plot shows the mitotic index of the cell cultures over time (compound A8, red bars; mock [1% DMSO], green bars) (***A***). Compound A8 inhibited spindle formation (α-tubulin) in HeLa cells characterized by detached sister chromatids (DAPI/CREST staining), as shown by confocal IF microscopy; nocodazole acted as a positive control. The antitubulin activity of compound A8 on human cells is reversible as the cells formed a mitotic spindle structure when the drug was washed out and the cells were released into fresh medium containing 10 µmol/l MG132. The scale bar represents 10 µm (***B***). Compound A8 inhibited tubulin assembly (upper panel) and triggered MT depolymerization *in vitro* (lower panel) (***C***). Compound A8 (50 µmol/l) mildly depolymerized taxol-stabilized MTs *in vitro*; nocodazole and maytansine (50 µmol/l) acted as positive controls. The bars represent standard errors (***D***).

**Figure 8:**
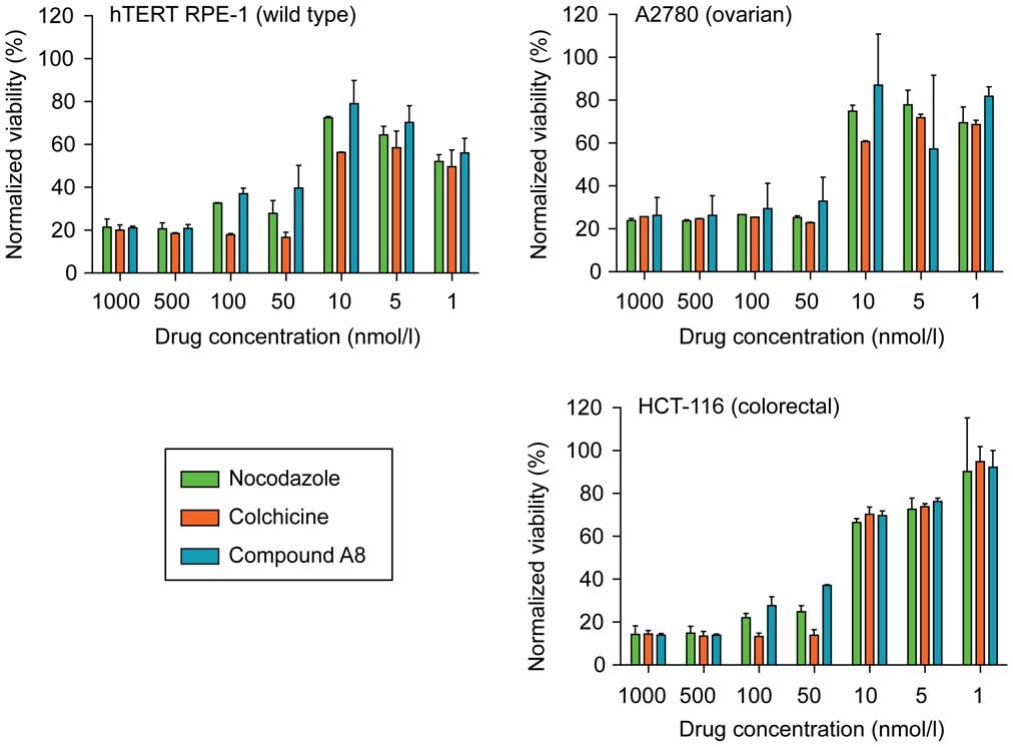
Furan-metoticas kill proliferating cells with an efficiency equaling that of established antitubulin drugs. Viability analysis of hTERT RPE-1 control cells and two adenocarcinoma cell lines following treatment with compound A8 (1–1000 nmol/l); nocodazole and colchicine acted as positive controls.

After confirming that our most active compound A8, inhibited spindle formation, depolymerized metaphase spindles, acted in a reversible manner, and triggered a mitotic arrest and death of HeLa cells (Fig. 7), we examined the efficacy of the compound on the growth of the A2780 (ovarian carcinoma) and HCT-116 (colorectal carcinoma) tumor cell lines. RPE, a telomerase-immortalized cell line derived from retinal pigment epithelium, served as the non-cancer control. Antitubulin agents colchicine and nocodazole were used as controls for drug activity. The RPE, A2780, and HCT-116 cells were efficiently killed by compound A8 and the control drugs. Importantly, the efficacy of compound A8 (IC_50_: 25 nmol/l) was similar to that of the established tubulin inhibitors (Fig. 8).

## Discussion

During the last twenty-five years antispindle drugs have been used with great success in the fight against cancer. However, as cancer cells are developing resistance against these drugs, there is an urgent need for compounds targeting alternative mitotic targets. As kinetochores orchestrate chromosome segregation and comprise >100 proteins, they are appealing mitosis-specific drug targets. The high antitumor activity of compounds inhibiting kinetochore regulators (Aurora kinase, PLK1) and the kinetochore-associated kinesin CENP-E supports the concept of targeting kinetochore function to eradicate proliferating cells [7], [9], [19]. The complexity of kinetochores, the lack of insight into intrakinetochore protein-protein contacts and protein-activity relationships, as well as the difficulty to produce kinetochore subunits in large quantities for use in *in vitro* screens has long hampered the conversion of structural kinetochore components into anticancer drug targets. Arguably the most intensely studied kinetochore subunit, both from a functional and structural point of view, is the outer kinetochore Ndc80 complex, which recruits the SAC and attaches the kinetochore structure to the MTs of the mitotic spindle [10], [12]. As the Ndc80 complex can be produced recombinantly in high quantity and because the recombinant complex is fully active as shown following injection in cells [12] we focused on this complex to screen for inhibitors of kinetochore-MT binding. Such inhibitors would leave sister chromatids detached from the spindle, leading to a robust SAC mediated arrest of the cells in mitosis. As mitotically arrested cells frequently undergo apoptotic death these drug would be potent eradicators of cancer cells characterized by uncurbed proliferation. In addition, we'd like to use these inhibitors to study how detached kinetochores prepare for kinetochore-spindle contact. Out of the 10,200 compounds that were screened, one molecule (compound B; 6-furan-2-yl-3-methyl-4-oxo-4,5,6,7,-tetrahydro-1*H*-indole-2-carboxylic acid tetrahydro-furan-2-ylmethyl ester) prevented binding of the Ndc80 complex to taxol-stabilized MTs by acting at the MT level. Indeed, the compound prevented MT binding not only of the Ndc80 complex but also of the MT plus-end tracking CLIP-170 protein, suggesting that it acted specifically towards the MTs. We confirmed this hypothesis and showed that the compound localized to the colchicine site at the αβ-tubulin interface. We believe that a conformational change in the MT polymers caused by binding of compound B to the colchicine pocket in the αβ-tubulin dimer may have prevented the association of the proteins with the MT surface. Importantly, colchicine-site agent nocodazole did not prevent the Ndc80 complex from binding to taxol-stabilized MTs, further arguing that compound B affects MT integrity in a unique manner. Of note, our screen also identified a second compound (4-5-2-1*H*-benzimidazol-2-yl-2-cyano-vinyl-furan-2-yl-benzoic acid), which specifically targeted the Ndc80 complex as it did not inhibit the MT binding of CLIP-170 nor of fluorescently labeled anti-tubulin antibodies (data not shown). The identification of this compound further validates the potency of our screen. Unfortunately, our study of the interaction between compound C and the Ndc80 complex has been complicated by the inability of the compound to enter cells. However, injecting the compound into HeLa cells significantly reduced the ability of the cells to align their sister chromatids to the metaphase plate, consistent with impaired kinetochore-spindle binding (De Wulf P. and DeLuca J.G., preliminary observations). We are currently manipulating the compound to make it cell permeable and are testing a battery of uncharged analogs to study their interaction with the Ndc80 complex, both *in vitro* and in cells.

Computer-assisted SAR analysis of compound B revealed that its furan-associated bicyclic nucleus harbors the antitubulin activity of the compound whereas its C2 side chain is important for overall activity, possibly by affecting the localization of the compound to the tubulin pocket. These analyses also led to the identification of compound analogs 20-fold more potent than compound B. Preliminary docking of the *R*- and *S*-enantiomers of compound B and its analogs onto the X-ray structures of N-deacetyl-N-(2-mercaptoacetyl)-colchicine and podophyllotoxin bound to tubulin [15], [16] revealed that the bulky C2-linked tetrahydrofuran and cyclohexane groups in compounds B and A6 may result in unfavorable ligand-protein and intra-ligand contacts, explaining why both compounds were the least active molecules, both *in vitro* and in cells. The analyses further showed that the stereoelectronic features of the *R*-enantiomers closely resemble those of colchicine, while the stereoelectronic properties of the *S*-enantiomers resemble those of colchicine-site drug podophyllotoxin. The *R*-enantiomers appear to interact with the tubulin colchicine pocket via their furan ring, while the *S*-enantiomers seem to localize to tubulin via their C2 side chains. To the best of our knowledge no other class of antitubulin compounds behaves in such a stereochemically unique manner. Separation of the compound isomers is in progress to determine whether the *R*- and *S*-forms show a different level of activity toward tubulin *in vitro* and in cells.

The most active of our colchicine site compounds kills tumor cells with an IC_50_ value of 25 nmol/l, a potency similar to that of spindle drugs used in the clinic. *In vivo* analyses will be performed to confirm efficacy in xenograft mouse models. So far colchicine site effectors (e.g. colchicinoids, combretastatins) have not been used in the clinic as they cause systemic toxicity. The classic colchicine site drugs are large, complex molecules that share a high level of structural similarity, e.g. most carry a tubulin-interacting trimetoxyphenyl group. However, novel colchicine site effectors that are structurally simple or chemotypically diverse may be the answer to the acute toxicity issues as illustrated by the stilbene colchicine derivatives, which are highly potent but minimally toxic agents [11]. Our antitubulin compounds represent a chemotypically unique set of colchicines site agents (e.g. they do not contain a trimetoxyphenyl group) and are some of the simplest antitubulin compounds reported to date, both in the scientific and patent literature. Their configuration and unique mode of stereoselective interaction with tubulin will allow for a better understanding of how antitubulin drugs work and will permit the model-driven chemosynthetic generation of derivatives with optimal activity and stability. Potent, non-toxic colchicine site analogs would represent much needed tools to battle cancer cells resistant to the currently used antispindle drugs.

## Materials and Methods

### Production of recombinant Ndc80^488^ kinetochore complex

Recombinant human Ndc80 complex (Ndc80^Bonsai^ complex) was produced in *Escherichia coli* using a bicistronic expression plasmid (Fig. 1A, [12]). Following transcription from the TAC promoter/operator by induction with 0.4 mmol/l IPTG (20°C, 15 h), GST-Nuf2-Spc24 and Ndc80-Spc25 polypeptides were generated, which dimerized intracellulary into the GST-Ndc80 complex. The complex was isolated from *E. coli* cell extract following incubation with glutathione beads. The complex was released from the beads with PreScission Protease (GE Healthcare Life Sciences) and contaminants were removed by gel filtration chromatography (Superdex 200, GE Healthcare Life Sciences). On average 4 mg of Ndc80 complex were obtained from 1 liter of cell culture. Next, 1 mg Ndc80 complex was labeled with 0.1 mg Alexa Fluor 488 C_5_-maleimide fluorophore (Invitrogen) at a 1.5-2 stoichiometry. The fluorescent complex (Ndc80^488^ complex) was separated from unreacted fluorophore by gel filtration chromatography (Fig. 1B) and was then used to screen for compounds inhibiting Ndc80^488^ complex-MT binding (Fig. 1C). The Ndc80^488^ complex is fully functional as it is recruited to and acts at kinetochores when injected into prometaphase HeLa cells [12].

### Screening for compounds that prevent binding of the Ndc80^488^ complex to MTs

MTs were polymerized (30 min, 37°C) from a 1∶10 mixture of rhodamine-labeled:unlabeled bovine brain αβ-tubulin (Cytoskeleton) in BRB-80 buffer (80 mmol/l PIPES pH 6.8, 1 mmol/l MgCl_2_, 1 mmol/l EGTA) and were then stabilized with 20 µmol/l taxol. The Ndc80^488^ complex (0.1 µmol/l) was incubated with 100 µmol/l compound (30 min, 24°C) in 96-well polypropylene plates (Eppendorf). The 10,000 compound DIVERset library (Chembridge) and 200 compounds from the Department of Medicinal Chemistry and Technologies, University of Rome La Sapienza were used in the screen. Next, the MTs (100 nmol/l) were added to the compound-Ndc80^488^ complex solution in a 1∶1 v/v ratio. After incubation (10 min, 24°C), the reaction mixtures were transferred to 96-well glass-bottom plates (Greiner Bio-one). Each well was imaged at 519 nm and 590 nm with an IX81 Olympus inverted wide-field microscope (Olympus). Total internal reflection fluorescence imaging was performed with an Olympus IX71 inverted wide-field microscope (Olympus). ImageJ was used for image processing (NIH, USA).

### Tubulin polymerization and MT stability analysis

Polymerization of 10 µmol/l αβ-tubulin in BRB-80 buffer was monitored turbidimetrically at 350 nm for 15 min in a temperature-controlled spectrophotometer (37°C; SpectraMax Plus^384^, Molecular Devices). To probe the effect of a compound on MT stability the molecule was added 5 min after initiation of tubulin assembly. The decrease in protein turbidity (350 nm) resulting from MT depolymerization was followed for 15 min at 37°C [13]. Quantitative comparisons of drug effects on tubulin assembly were also examined in 0.8 M glutamate with a 15 min drug-tubulin preincubation prior to the addition of GTP [14]. Tubulin assembly was monitored for 20 min at 30°C in Beckman DU7400/7500 spectrophotometers, and the compound concentration inhibiting tubulin assembly by 50% was determined.

### [^3^H]colchicine competition experiments

Tubulin binding of [^3^H]colchicine was measured by the DEAE-cellulose filter method [20]. Reaction mixtures contained 1.0 µmol/l αβ-tubulin, 1.0 mol/l monosodium glutamate, 0.1 mol/l glucose-1-phosphate, 1.0 mmol/l MgCl_2_, 1.0 mmol/l GTP, 0.5 mg/ml BSA, 5% DMSO, 5 µmol/l [^3^H]colchicine, and inhibitor at 1, 5, 20 or 50 µmol/l. These reaction conditions strongly stabilize the colchicine-binding activity of tubulin [21].

### Compound-based depolymerization of taxol-stabilized MTs

Taxol-stabilized MTs were prepared and diluted as described for the Ndc80^488^ binding experiments. Varying compound concentrations in 10 µl of DMSO were added to 490 µl of diluted MTs at 37°C. Following incubation (30 min, 37°C), 450 µl of each mixture was centrifuged (10 min; 30,000 rpm; 37°C) in a Beckman Optima TLX centrifuge (TLA120.1 rotor). Pellets were dissolved in 25 µl of 8 mol/l urea, and the protein content of 20 µl was compared with that from pellets formed without compound.

### Viability analysis of adenocarcinoma cells

Viability analysis in the presence of antitubulin drugs was performed on hTERT RPE-1 (ATCC), HCT-116 (ATCC), and A2780 (EACC) cell lines. Cell lines were authenticated by PCR [22]. Absence of eight commonly encountered mycoplasma species was confirmed by PCR [23]. Experiments were performed with freshly thawed cells, which were seeded in 96-well MICROTEST tissue culture plates (Falcon) at ∼500 cells/well and grown at 37°C. After 6 h, nocodazole, colchicine or compound A8 were added (1–1000 nmol/l). The cells were incubated for 3 d at 37°C and then assayed for viability (CellTiter 96 Assay, Promega).

### Immunofluorescence (IF) imaging

G1/S synchronization with 2.5 mmol/l thymidine was carried out for 16 h. IF imaging (Leica TCS SP2 confocal microscope) was performed on cells fixed with 4% paraformaldehyde, permeabilized with 0.1% Triton X-100 and treated with 4% BSA. The cells were incubated with appropriate antibodies diluted in 4% BSA in PBS. Primary antibodies used in IF imaging were mouse monoclonal antiNdc80 antibody (1∶1000, Genetex Clone 9G3.23), CREST serum (1∶50, Antibodies Incorporation) and mouse monoclonal anti-α-tubulin antibody (1∶2000, Sigma-Aldrich Clone B512). Secondary antibodies used were Alexa Fluor 488 (1∶150), Cy3 (1∶400) and Cy5 (1∶50) (Jackson Immunoresearch).

## Supporting Information

Figure S1 Fragments used to search chemical libraries for compound analogues. Fragments of compound B (6-Furan-2-yl-3-methyl-4-oxo-4,5,6,7-tetrahydro-1H-indole-2-carboxylic acid tetrahydro-furan-2-ylmethyl ester), encircled in blue and red, that were used in the computer-assisted search of compound libraries.
(1.46 MB TIF)
