# Supplementary figures and images for: A Screen for Kinetochore-Microtubule Interaction Inhibitors Identifies Novel Antitubulin Compounds
Source: PLoS One. 2010 Jul 15;5(7):e11603. doi: 10.1371/journal.pone.0011603 (PMC2904697; doi:10.1371/journal.pone.0011603)

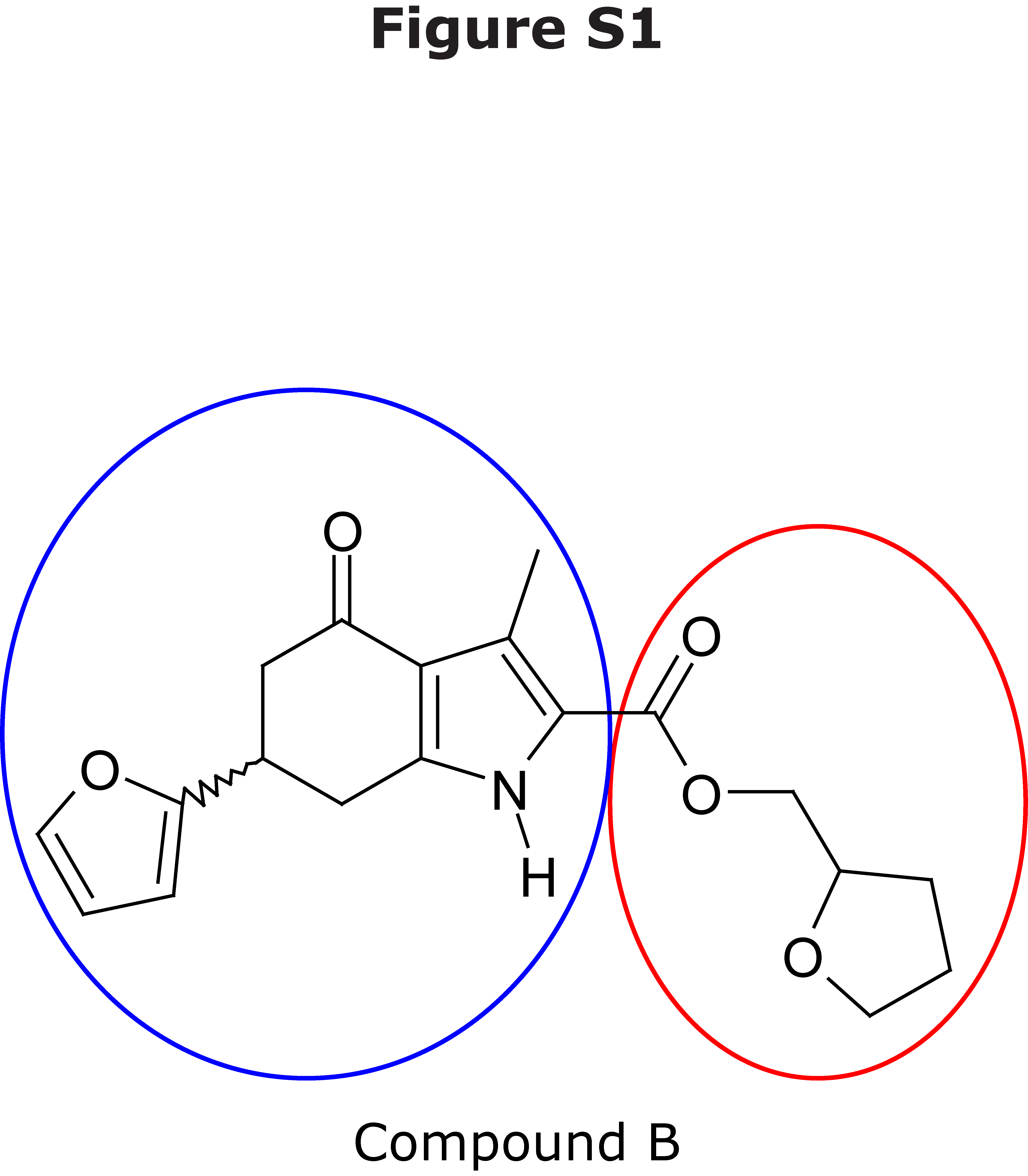

Supplement: Figure S1 — Fragments used to search chemical libraries for compound analogues. Fragments of compound B (6-Furan-2-yl-3-methyl-4-oxo-4,5,6,7-tetrahydro-1H-indole-2-carboxylic acid tetrahydro-furan-2-ylmethyl ester), encircled in blue and red, that were used in the computer-assisted search of compound libraries. (1.46 MB TIF) [file pone.0011603.s001.tif]
